# Balloon surface temperature–controlled ablation using a second-generation radiofrequency HotBalloon: an *in vivo* feasibility study

**DOI:** 10.1093/europace/euad340

**Published:** 2023-11-09

**Authors:** Yasutoshi Shinoda, Hiro Yamasaki, Nobuyuki Murakoshi, Tsunesuke Kohno, Teiichi Yamane, Kazutaka Aonuma, Tomoko Ishizu

**Affiliations:** Department of Cardiology, Faculty of Medicine, University of Tsukuba, 1-1-1 Tennodai, Tsukuba, Ibaraki 305-8575, Japan; Department of Cardiology, Faculty of Medicine, University of Tsukuba, 1-1-1 Tennodai, Tsukuba, Ibaraki 305-8575, Japan; Department of Cardiology, Faculty of Medicine, University of Tsukuba, 1-1-1 Tennodai, Tsukuba, Ibaraki 305-8575, Japan; Department of Cardiology, Nagano Chuo Hospital, Nagano, Japan; Department of Cardiology, Faculty of Medicine, Tokyo Jikeikai Medical University, Minato-ku, Tokyo, Japan; Department of Cardiology, Faculty of Medicine, University of Tsukuba, 1-1-1 Tennodai, Tsukuba, Ibaraki 305-8575, Japan; Department of Cardiology, Mito Saiseikai General Hospital, Mito, Ibaraki, Japan; Department of Cardiology, Faculty of Medicine, University of Tsukuba, 1-1-1 Tennodai, Tsukuba, Ibaraki 305-8575, Japan

**Keywords:** Animal study, Atrial fibrillation, Balloon surface temperature, Catheter ablation, Radiofrequency HotBalloon

## Abstract

**Aims:**

The first-generation radiofrequency HotBalloon (RHB) is a size-adjustable single-shot device used in atrial fibrillation. The energy output is determined by its central temperature and not by its balloon surface temperature (BST), thus limiting its efficacy and safety. Therefore, a second-generation RHB was developed to monitor BST and enable BST-controlled ablation. This animal study aims to evaluate the accuracy of a newly developed BST-monitoring system and validate the optimal BST for ablation.

**Methods and results:**

In Protocol 1, thermocapsules were attached to the superior vena cava (SVC) epicardium. The accuracy of BST monitoring was examined during SVC isolation. In Protocol 2, the efficacy and safety of different BST-controlled ablations were examined. In the acute model, electrophysiological and pathological findings were assessed after energy applications with BST at 51, 54, 57, and 60°C. In the chronic model, the lesion durability and pathological findings were assessed 8 weeks after BST-controlled ablation (57 and 60°C). A significant positive correlation was found between the epicardial temperature and the BST-monitoring value (*r* = 0.98). In the acute model, all target veins were electrically isolated with BST-controlled ablation at ≥57°C (18/18, 100%). In the chronic model, durable lesions were observed in all veins at 60°C, while 44% of the veins showed reconnection at 57°C. In both pathological analyses, significantly greater lesions were observed at 60°C than at 57°C. There were no significant differences in adverse events between the two groups.

**Conclusion:**

Balloon surface temperature–controlled ablation at 60°C using the second-generation RHB may be optimal for creating durable lesions without compromising safety.

## Graphical Abstract

**Graphical Abstract:**
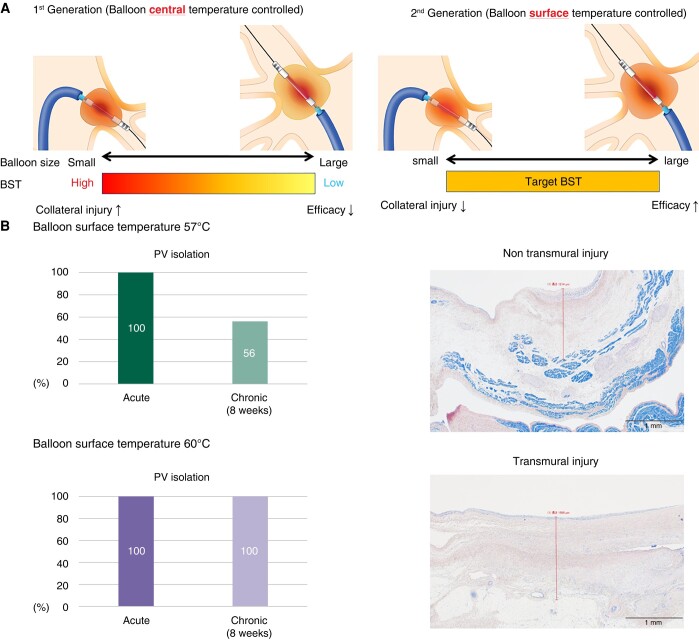
Principle of balloon surface temperature–controlled ablation and its efficacy. (*A*) Principles of the first- and second-generation radiofrequency HotBalloon (RHB) and their impact on the efficacy and safety of energy applications. (*B*) Electrical isolation rate and representative pathological images of RHB ablation with balloon surface temperatures of 57 and 60°C. BST, balloon surface temperature.

**What’s new?**

- Lesion formation using balloon surface temperature (BST)–controlled radiofrequency HotBalloon ablation (RHB) of the myocardium was enhanced depending on the BST.
- Balloon surface temperature–controlled ablation at 60°C is a feasible method for acute and chronic pulmonary vein (PV) isolation without increasing collateral damage and PV stenosis.
- Balloon surface temperature–controlled ablation strengthens the advantage of size-adjustable RHB regardless of balloon size and may contribute to improved acute success rates.

## Introduction

**Figure 1:**
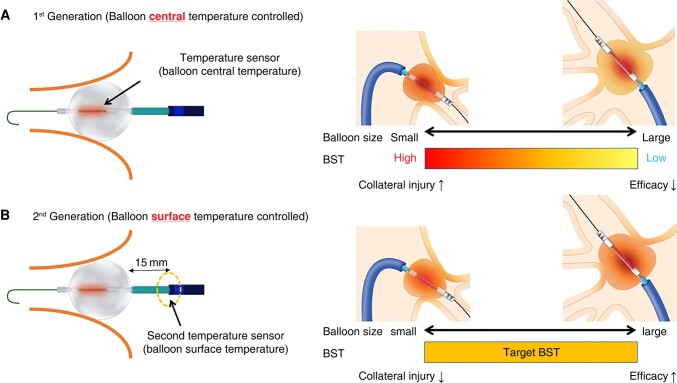
Principles of the first- and second-generation RHB and their impact on the efficacy and safety of energy applications. (*A*) With the first-generation RHB, energy output is regulated by the balloon’s central temperature. (*B*) With the second-generation RHB, an additional temperature sensor was attached 15 mm from the proximal end of the balloon, and BST-controlled ablation became available. BST, balloon surface temperature.

First-generation radiofrequency HotBalloon (RHB; SATAKE HotBalloon, Toray Industries, Inc., Tokyo, Japan) catheter ablation is a feasible treatment for paroxysmal atrial fibrillation (AF).^1–4^ The RHB catheter consists of a size-adjustable high-compliance balloon (25–33 mm) that enables better contact with various pulmonary vein (PV) anatomies with a system that regulates power output to maintain the target balloon central temperature (BCT) up to 73°C and an agitation pump that mixes the inner injected fluid. Although a uniform balloon surface temperature (BST) is achieved by agitating the inner balloon fluid, it is affected by the distance between the balloon’s central coil and the balloon surface. When the target BCT was constant, the balloon injection volume and BST showed an inverse correlation. During the clinical trial, energy was delivered with a smaller balloon injection volume. A higher BST and energy application inside the PV ostium resulted in a high acute success rate and a high incidence of collateral injuries.^5^ To avoid these complications, an energy application with a larger balloon injection volume was adopted after the device was introduced to the market.^4,6^ The acute success rate in these studies was lower than that in clinical trials, which was speculated to be attributed to the lower BST using a larger balloon injection volume (*Figure 1A*).

A simulated biological model and computer-aided engineering analysis revealed that temperature sensors attached at 5 and 25 mm from the proximal end of the balloon could accurately estimate the BST by measuring the temperature of the diluted contrast media sucked from the balloon into the catheter tube regardless of whether the balloon was coaxial or non-coaxial to the PV.^7^ Based on this finding, a second-generation RHB system with an additional intra-tube temperature sensor that is attached at 15 mm from the proximal end of the balloon for monitoring the BST was developed to overcome the limitations of the first-generation RHB system. A recent clinical study using the second-generation RHB demonstrated that a BST of >58.7°C predicted acute PV isolation.^8^ However, energy delivery was controlled automatically to achieve a pre-specified BCT, and the feasibility of BST-controlled ablation was not tested. Balloon surface temperature–controlled ablation is expected to improve both efficacy and safety, thereby strengthening the advantage of the size-adjustable RHB (*Figure 1B*). Accordingly, we conducted an *in vivo* study to examine the accuracy of BST monitoring of the second-generation RHB and validate the optimal BST to achieve durable lesions without compromising safety.

## Methods

**Figure 2:**
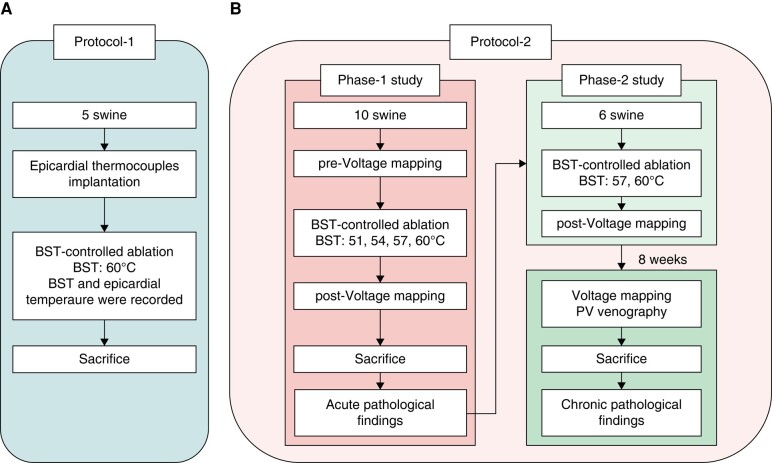
Study design of Protocols 1 and 2. (*A*) Study design of the Protocol 1 verifying the accuracy of the BST-monitoring system. (*B*) Study design of the Protocol 2. The left diagram shows the Phase 1 study evaluating acute electrophysiological and pathological findings. The right diagram shows the Phase 2 study evaluating chronic electrophysiological and pathological findings. BST, balloon surface temperature; PV, pulmonary vein.

This study used closed-chest swine and was divided into two protocols. In Protocol 1, the accuracy of the BST-monitoring system was verified. In Protocol 2, BST-controlled ablation was performed using different target BSTs to evaluate acute electrical isolation and lesion formation (Phase 1). Following the acute study, lesion durability, and collateral injuries after 8 weeks were assessed (Phase 2). The study designs for the two protocols are summarized in *Figure 2* (*A*: Protocol 1; *B*: Protocol 2). In both studies, a second-generation RHB was used for all ablation procedures.

### Animal preparations

All animal experimental procedures were approved by the Institutional Animal Experiment Committee of the University of Tsukuba and the Institutional Animal Care and Use Committee of IVTeC Co., Ltd (NRT-191001, NRT-20120, and NRT-210402). Experiments were conducted in accordance with the Guide for the Care and Use of Laboratory Animals published by the US National Institutes of Health and the Fundamental Guideline for Proper Conduct of Animal Experiment and Related Activities in Academic Research Institutions under the jurisdiction of the Ministry of Education, Culture, Sports, Science and Technology of Japan. Twenty-one Yorkshire domestic swine (mean bodyweight: 42.7 ± 1.8 kg) were utilized. All animals received antiplatelet therapy 1 day prior to the procedure. General anaesthesia was maintained with isoflurane, and oxygen was delivered through a precision vaporizer and a circular absorption breathing system. Bilateral femoral venous access was obtained, and two 8F-long sheaths (SL0; Abbott, Chicago, IL, USA) were positioned in the right atrium (RA) under fluoroscopic guidance. Heparin was administered intravenously to achieve an activated clotting time of >250 s during the procedure.

### HotBalloon ablation using a second-generation radiofrequency HotBalloon system

A single transseptal puncture was performed, and two 8F-long sheaths were guided to the left atrium (LA). A circular mapping catheter (Optima; Abbott, Plymouth, MN, USA) was inserted into the LA for detailed voltage mapping using a three-dimensional mapping system (Ensite NavX; Abbott). An 8F-long sheath was replaced with a J-tip guidewire (Spring Guide Wire; Toray Medical Co., Ltd, Tokyo, Japan) for a 17F deflectable guiding sheath (Treswaltz; Toray Industries, Inc., Tokyo, Japan). Subsequently, the RHB catheter was advanced into the LA using a 17F guiding sheath. The balloon was advanced to the ostium of the target vein and inflated with a 50% mixture of saline and contrast media. The curve of the deflectable guiding sheath was adjusted to achieve the coaxial position of the RHB to the antrum of the target veins. Subsequently, target vein occlusion was confirmed using a selected PV angiogram. Once the BST reached the target value, a single energy application was delivered to the bilateral PVs and superior vena cava (SVC) for 180 s, with no additional energy application. The current generator automatically titrates the energy output to maintain the target BCT but not the BST. In this study, the energy output was manually regulated to maintain the targeted BST instead of the BCT to evaluate the efficacy and safety of BST-controlled ablation. Voltage mapping was performed before and after RHB ablation to evaluate the electrical isolation of the targeted veins. After energy application, the RHB was extracted, and the formation of balloon surface thrombosis was assessed.

### Protocol 1: validation of the balloon surface temperature–monitoring system

**Figure 3:**
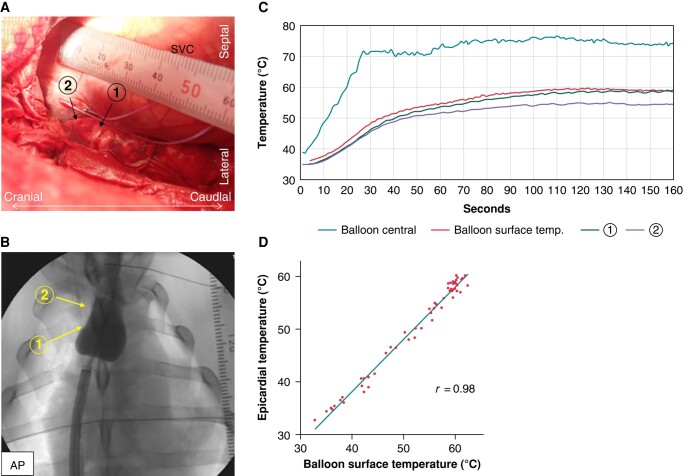
Method and result of the Protocol 1. (*A*) Thermocouple implantation in the epicardial surface of the SVC and RA junction. (*B*) Fluoroscopy image of energy application at the SVC–RA junction. Two thermocouples were positioned just opposite the RHB (arrow). (*C*) Representative scatterplots of the epicardial temperature, temperature recorded at the BST-monitoring system, and BCT during energy application. (*D*) Scatterplots showing the correlation between the epicardial temperature of the SVC–RA junction and the BST. AP, anterior-posterior; SVC, superior vena cava.

Five swine were included in this study. Two thermocouples (Physitemp Instruments, Inc., Clifton, NJ, USA; time constant: 0.1 s, diameter: 0.64 mm, accuracy: ±0.1°C) were surgically implanted at the epicardial surface of the SVC–RA junction to monitor the epicardial temperature (*Figure 3A* and *B*). A single energy application was delivered to the SVC for 180 s with a target temperature of 60°C. The temperature of the SVC epicardium served as a control for the BST-monitoring system. The epicardial temperature, the temperature recorded using the BST-monitoring system, the BCT, and the radiofrequency output were recorded every 0.1 s. The BST and temperature of the thermocouple closest to the RHB surface at the SVC–RA junction were plotted every 10 s to evaluate the correlation between SVC epicardial temperature and the BST (*Figure 3C*).

### Protocol 2: feasibility study of balloon surface temperature–controlled ablation

We conducted a two-phase study to validate the optimal BST for energy application. The Phase 1 study was designed to determine the acute efficacy of the BST-controlled energy application using different BST targets. Subsequently, a Phase 2 study was designed to evaluate the chronic effect of the BST-controlled energy application using the BST values tested in the Phase 1 study.

### Phase 1 study: acute evaluation (determining the optimal balloon surface temperature)

The acute effects of BST-controlled ablation were evaluated in 10 swine (10 left PVs, 10 right PVs, and 10 SVCs) with different BSTs. In this study, four different target BSTs (51, 54, 57, and 60°C) were used. Each targeted vein was ablated in a random fashion using a different target BST for 180 s once the BST reached the target value. Voltage mapping was performed before and after each energy application to assess the electrical isolation of the target veins. All swine were sacrificed for gross anatomical and histological examination.

### Phase 2 study: chronic evaluation (lesion durability and safety)

Six swine were included in this Phase 2 study. Based on the Phase 1 study, target veins were ablated only with a BST of 57 or 60°C. After ablation, a voltage map was obtained to assess acute electrical isolation. Eight weeks after the procedure, an electrophysiological study was repeated to assess the lesion’s durability. Target vein stenosis was evaluated using the venography of each vein, and severe stenosis was defined as a >70% reduction in the diameter of the targeted veins. Finally, all the swine were sacrificed for gross anatomical and histological examination.

### Gross anatomy and histological examination

**Figure 4:**
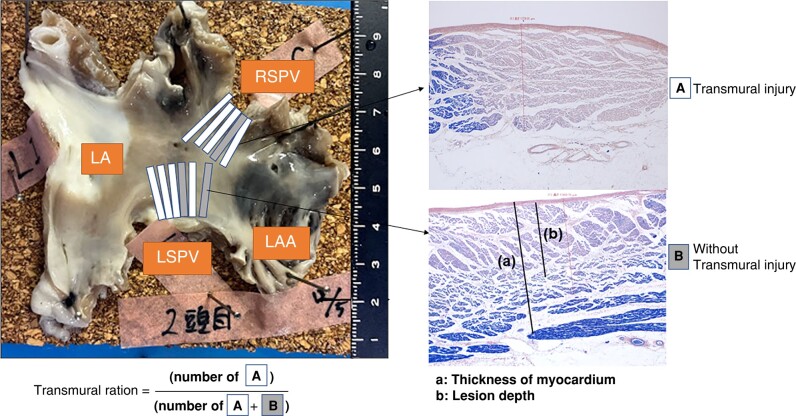
Histological illustrations of the lesions using PTAH staining. The white and grey boxes show a longitudinal section of the LSPV and RSPV. The white box (*A*) shows transmural injury, and the grey box (*B*) shows non-transmural injury. LA, left atrium; LAA, left atrial appendage; LSPV, left superior pulmonary vein; RSPV, right superior pulmonary vein.

Prior to heart removal, tissues surrounding the ablation sites were examined for collateral damage, with particular attention paid to the oesophagus, bronchi, aorta, pulmonary artery, diaphragm, and phrenic nerves. The hearts were exposed and excised and then submerged with the attached vessels in a suitably sized container filled with 10% neutral-buffered formalin. The LA was opened along the roof and inspected grossly, and tissue blocks containing each PV were dissected from the LA. The veins were sliced into serial parallel sections (*Figure 4*) for histological examination. Each PV tissue block was stained with haematoxylin, eosin, and phosphotungstic acid-haematoxylin (PTAH). The transmural ratio (segments with transmural injury/resected slides for each vein)^5^ and the depth of the ablated lesion (lesion depth) were evaluated.

### Statistics analysis

Continuous variables were expressed as means ± standard deviation. Categorical variables were expressed as numbers and percentages and compared using *χ*^2^ or Fisher’s exact tests, as appropriate. The Pearson rank correlation test was used to analyse the correlation between the BST and the epicardial temperature in the thermocouple. All statistical analyses were performed using the JMP software version 13.0 (SAS Institute Inc.), with statistical significance set at *P* < 0.05.

## Results

### Protocol 1: validation of the balloon surface temperature–monitoring system

The recorded temperatures and RHB-related data are presented in *Table 1*. The temperature of the BST-monitoring system reached the target temperature within 61.2 ± 11.9 s after the energy application. A significant positive correlation was observed between the epicardial surface temperature close to the balloon and the value recorded using the BST-monitoring system (*r* = 0.98, *Figure 3D*). None of the epicardial tissue temperatures exceeded the BST during the RHB energy delivery. Representative scatterplots of the BST, BCT, and epicardial temperature are shown in *Figure 3C*.

**Table 1:** Procedural data regarding SVC isolation

SVC ablation	
Balloon injection volume, mL	12.4 ± 0.8
Time to targeted temperature, s	61.2 ± 11.9
Mean epicardial tissue temperature, °C	58.3 ± 0.6
Mean balloon surface temperature, °C	60.0 ± 1.2
Mean balloon central temperature, °C	70.4 ± 0.8
Mean RF power, W	81.1 ± 10.1

Data are presented as mean ± SD. RF, radiofrequency; SVC, superior vena cava

### Protocol 2: phase 1 study (acute evaluation)

**Figure 5:**
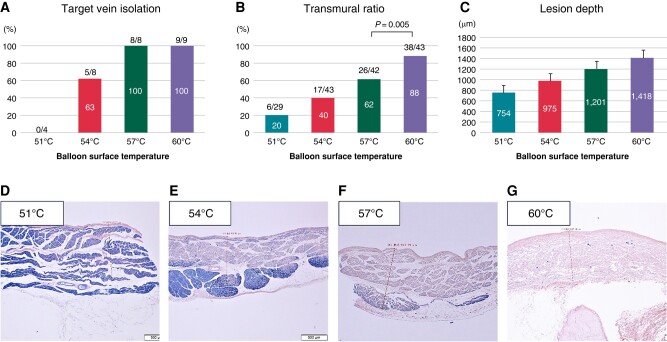
Acute electrophysiological and pathological findings after energy application using four different BSTs. (*A*) Acute electrical isolation rate of target veins. (*B*) Transmural ratio. (*C*) Lesion depths. Representative resected slides stained by PTAH staining (*D*: 51°C, *E*: 54°C, *F*: 57°C, *G*: 60°C). Of note, energy application with a higher BST resulted in uniform myocardial injury. An apparent positive association between BST and lesion depth was observed, but actual lesion depth was difficult to measure when the transmural lesion was achieved. Hence, a statistical comparison of lesion depth between each BST was not performed.

The electrophysiological and histological outcomes of the Phase 1 study are summarized in Supplementary material online, *Table S1* and *Figure 5*. One right superior PV (RSPV) precluded complete PV occlusion because of the proximity between the transseptal puncture site and the PV ostium and was, therefore, excluded from the analysis. Accordingly, 29 veins [10 left superior PVs (LSPVs), 9 RSPVs, and 10 SVCs] were evaluated. Acute electrical isolation was obtained in all PVs and SVCs ablated with a BST of ≥57°C (17/17, 100%) but in five of eight (63%) PVs/SVCs with a BST of 54°C and none with a BST of 51°C (*Figure 5A*). All energy application was achieved using a current generator without exceeding the maximal limits of radiofrequency power (150 W) and BCT (73°C). Thrombus formation was not observed on the balloon surface in any BST setting. Acute histological examination demonstrated that the transmural ratio (*Figure 5B*) with a BST of 60°C was significantly higher than that at 57°C (88.4 vs. 61.9%, *P* = 0.005). Additionally, there was a clear positive association between the BST and the lesion depth (*Figure 5C* and Supplementary material online, *Table S1*). Representative images are shown in *Figure 5D*–*G*. As the BST increased, deeper uniform lesions were created. Although PVs ablated with a BST of ≥57°C were electrically isolated, transmural lesions were observed only in veins ablated with a BST of 60°C. Gross anatomy revealed encircling lesions without thrombus or charring and no evidence of collateral injury.

### Protocol 2: phase 2 study (chronic efficacy evaluation)

**Figure 6:**
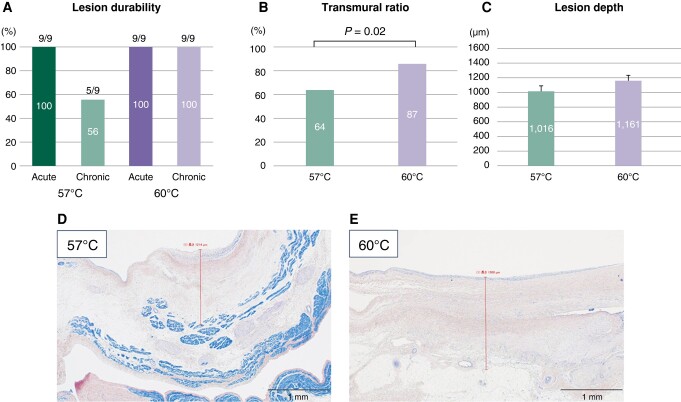
Chronic electrophysiological and pathological findings comparing energy application using BSTs of 57 and 60°C. (*A*) Electrical isolation rate during the ablation and after 8 weeks. Energy application with a BST of 60°C created durable lesions, while energy application with a BST of 57°C resulted in a 44% reconnection rate. (*B*) The transmural ratio was significantly greater after energy application at a BST of 60°C. (*C*) Lesion depths. (*D* and *E*) Representative resected slides of the chronic phase stained by PTAH staining.

**Figure 7:**
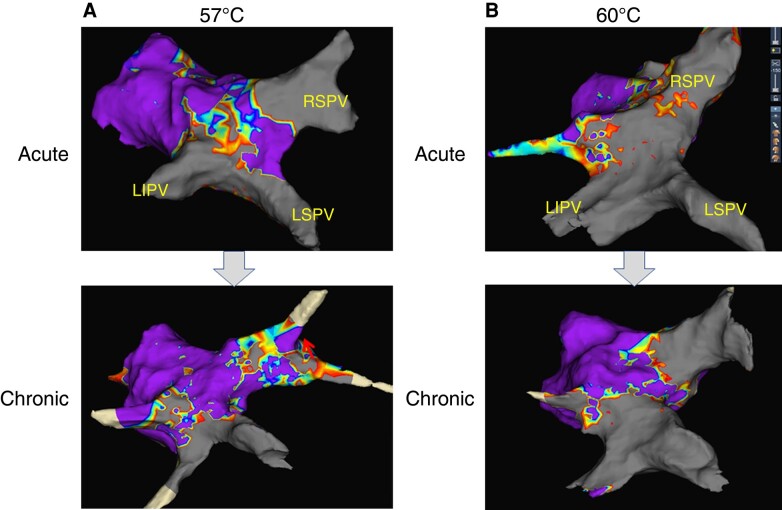
Representative voltage map in the LA and PV for acute and chronic evaluation. (*A*) Voltage maps of ablation with a BST of 57°C in the acute and chronic phases. (*B*) Voltages map of ablation with a BST of 60°C in the acute and chronic phases. For details, see text. LIPV; left inferior pulmonary vein; LSPV, left superior pulmonary vein; RSPV, right superior pulmonary vein.

The electrophysiological and histological outcomes of the Phase 2 study are summarized in Supplementary material online, *Table S2* and *Figure 6*. Acute electrical isolation was obtained in all veins with BSTs of 57 and 60°C. All swine survived for 8 weeks after RHB ablation and underwent chronic evaluation. The chronic electrophysiological findings are shown in *Figure 6A*. Lesion durability after energy application with a BST of 60°C was 9/9 (100%), whereas reconnection was observed in four of nine target veins (44%; 2 RSPVs, 1 LSPV, and 1 SVC) ablated with a BST of 57°C. The representative voltage maps are shown in *Figure 7*. Although acute bilateral PV isolation was achieved with a BST of 57°C, reconnection was observed at the roof and carina of the right PV (*Figure 7A*). The resected slide corresponding to the site of electrical reconnection revealed an incomplete transmural lesion (*Figure 6D*). In contrast, durable isolation was confirmed with a BST of 60°C (*Figure 7B*), and the pathological assessment demonstrated transmural injury in all resected slides (*Figure 6E*). The transmural ratio (*Figure 6B*) with a BST of 60°C was significantly higher than that with a BST of 57°C (87 vs. 64%, *P* = 0.02). Compared with the acute phase, a greater lesion depth with a BST of 60°C was consistently observed compared with a BST of 57°C (*Figure 6C* and Supplementary material online, *Table S2*).

### Protocol 2: phase 2 study (chronic safety evaluation)

There was no significant difference in target vein stenosis between BSTs of 57 and 60°C [1/9 (11%) vs. 0/9 (0%), *P* = 0.30], but one severe RSPV stenosis developed after energy application with a BST of 57°C (see Supplementary material online, *Figure S1*). A mild oesophageal thickening was observed in two cases (one each, after BST with 57 and 60°C), but no histological change suggested oesophageal ulceration. A pericardial adhesion was observed in one case with a BST of 60°C. No collateral injuries were observed.

## Discussion

### Main findings

This *in vivo* study validated the accuracy of a BST-monitoring system equipped with the second-generation RHB, as well as the feasibility of BST-controlled ablation. The main findings of this study are summarized as follows: First, the inner balloon temperature recorded at the additional thermo-sensor attached to the proximal part of the second-generation RHB showed a strong positive correlation with the SVC epicardial temperature close to the RHB surface. This result suggests that the value recorded at the additional thermo-sensor in the second-generation RHB would approximate the BST during energy applications. Secondly, lesion formation in BST-controlled ablation of the myocardium was enhanced depending on the BST. Notably, electrical isolation did not always correspond to pathological lesion formation, and a BST of 60°C was required for greater transmural lesions. Thirdly, BST-controlled ablation at 60°C showed significantly better lesion durability than that with a BST of 57°C. Finally, collateral injury was not increased with BST-controlled ablation at 60°C. These findings indicated that a BST of 60°C was well-balanced in terms of efficacy and safety; thus, it may become the target BST for BST-controlled ablation.

### Balloon surface temperature–monitoring system

With the first-generation RHB, an empirical protocol guided by BCT-controlled ablation was used, and the actual BST, which directly affects lesion formation, was unknown. A second-generation RHB system with an additional temperature-monitoring sensor placed 15 mm away from the catheter shaft was designed based on a simulated biological model and computer-aided engineering analysis. This enabled temperature recording, which was expected to approximate the actual BST.^5^ This *in vivo* study demonstrated that the BST measured with the additional temperature-monitoring sensor showed a strong positive correlation with the SVC epicardial temperature close to the balloon surface. Accordingly, BST-controlled ablation is expected to improve the efficacy and safety and enhance the advantages of a size-adjustable single-shot balloon device (*Figure 1B*).

### Efficacy of balloon surface temperature–controlled radiofrequency HotBalloon ablation

Heat conduction is a crucial element in lesion formation using the RHB system, and both the energy application time and the BST play important roles.^5,9^ A randomized controlled study using a smaller balloon injection volume resulted in a high acute isolation rate (98%) but with a considerable rate of severe PV stenosis and phrenic nerve injury. After the clinical trial, a balloon injection volume of >10 mL was generalized to avoid PV stenosis and phrenic nerve injury. After approval of the device in Japan, several registries using a larger balloon injection volume reported a lower acute isolation rate (51–73%) with the RHB and required touch-up ablation.^2,8,10^ The lack of an established ablation protocol and technique, limited experience, and non-coaxial position of the RHB were speculated as reasons for the low acute PV isolation rate.^2,7^ Most importantly, BST-controlled ablation is not technically available with the first-generation RHB, which directly affects lesion formation. The power was titrated to maintain the target BCT and the agitation pump facilitated to achieve a uniform BST during energy application. However, several conditions such as a greater distance from the balloon central coil to the balloon surface and a greater peri-balloon leak result in a lower BST that would lower the PV isolation rate.

Nakahara *et al*. demonstrated an acute success rate of second-generation RHB ablation using BST monitoring. They reported that a BST of >58.7°C was the cut-off value for predicting the acute success rate.^8^ Recently, Fukunaga *et al*.^11^ identified a median BST of 58°C as the optimal value for achieving acute PV isolation. However, the energy was delivered via BCT-controlled ablation rather than through BST-controlled ablation. In addition, the association between BST, lesion durability, and chronic outcomes has not yet been explored. Our results demonstrate a discrepancy between acute and chronic findings. Despite acute electrical isolation being achieved with BST-controlled ablation at 57°C, 44% of the target veins demonstrated reconnection. In contrast, durability after BST-controlled ablation at 60°C was high, and a pathological study showed significantly greater lesion formation at 60°C. Based on the results, a greater transmural ratio suggests electrical isolation. In contrast, a greater transmural ratio is not a requirement for electrical isolation. Target veins were all isolated after energy application with a BST of 57°C, while the transmural ratio was 62%. These findings are consistent with the fact that electrical PV isolation can still be achieved with non-transmural lesions when conductive heating elicits tissue oedema and reversible cell damage.^12,13^ Those insufficient lesions can lead to PV reconnection in the chronic phase. Because the chronic phase evaluation was conducted 8 weeks later in this study, the sites with incomplete transmural lesions could potentially contribute to reconnection during further long-term follow-up.

### Chronic safety of balloon surface temperature–controlled radiofrequency HotBalloon ablation

Pulmonary vein stenosis and phrenic nerve injury are two major concerns during balloon-based ablation. Animal studies assessing lesions after RHB ablation showed that a higher BCT and longer energy application time are associated with a deeper lesion,^7^ while marked fibrin deposition^5^ or PV stenosis may develop in patients with a hyper-reaction to fibrosis.^14^ In a clinical trial, the first-generation RHB with a small balloon injection volume was used, and severe PV stenosis was found in 5.2% of the cases. Recently, a single-centre study reported that the incidence rates of moderate and severe PV stenosis were 8.3 and 3.6%, respectively.^15^ The authors speculated that a longer energy application was responsible for the PV stenosis. Another study reported severe stenosis in the RSPV after energy application for 210 s, and the rate of PV stenosis was lower in the inferior PV after energy application for 120 s.^16^ In this animal study, one RSPV stenosis was observed but developed after energy application at 57°C and not at 60°C. Retrospectively analysed, the distal balloon portion was inside the right PV ostium to maintain the balloon position during energy application and may lead to severe PV stenosis. Our findings suggest that BST-controlled ablation at 60°C was well-balanced in terms of safety and efficacy.

### Clinical implications

One of the unique advantages of the RHB is that it is a size-adjustable single-shot balloon device that can be used with various PV anatomies. A previous study demonstrated that 8.1% of energy applications required a balloon injection volume ≥15 mL (estimated balloon diameter, 30 mm).^2^ Energy application using a larger balloon size leads to a wider PV isolation area and is expected to improve the clinical outcomes of AF ablation. When BST-controlled RHB ablation is technically feasible, it improves the acute success rate regardless of the balloon size and reduces collateral damage.

### Limitations

This study has the following limitations: First, the effects of different energy application durations on lesion formation were not evaluated. In a previous animal study,^5^ transmural lesions were increased in a time-dependent manner with a BCT of 70°C, and 180 s of energy application showed the greatest transmural lesions. As the objective of the study was to elucidate the optimal BST to achieve durable lesions, a uniform energy application time (180 s) was adopted in the study. Validation of the optimal energy application time for BST-controlled ablation is required. Secondly, ablation with a BST of >60°C was not tested. In a clinical trial and multicentre registry study,^1,6^ BCT-controlled ablation with a target BCT of 70°C was adopted. Balloon surface temperature monitoring was not technically available, but a previous animal study suggested that the BST is approximately 10°C lower than the BCT. Therefore, we assumed that PV stenosis and phrenic nerve injury in the clinical trial and multicentre registry study may have developed after energy application with a BST of around 60°C. Consequently, we considered that it would be appropriate to begin our animal study by examining the efficacy and safety of energy applications with a maximum BST of 60°C. While the target BST of 60°C showed balanced results, BSTs over 60°C were not tested. Thirdly, automated BST-controlled ablation was not available, and the target BST was maintained by manually adjusting the BCT. In the study, BST was controlled within 60.0 ± 1.2°C; thus, we believe that manual BST-controlled ablation in this study had a limited impact on the result. Fourthly, the lack of apparent collateral damage in the study should be interpreted carefully as the number of animals was relatively small. Furthermore, higher output and temperature during radiofrequency ablation are associated with the formation of microemboli.^17,18^ Therefore, higher BSTs may generate asymptomatic microthrombi formation, which was not evaluated in our study. Further clinical studies using automated BST-controlled ablation with a larger number of subjects are required to establish an optimal ablation protocol. Finally, an apparent positive association between BST and lesion depth was observed, but actual lesion depth was difficult to measure when the transmural lesion was achieved. Hence, a statistical comparison of lesion depth between each BST was not performed.

## Conclusions

After accurately evaluating the BST using the second-generation RHB system, we conclude that BST-controlled ablation at 60°C may be optimal for creating a transmural lesion without increasing collateral damage or PV stenosis.

## Supplementary Material

euad340_Supplementary_Data

## Data Availability

The data that support the findings of this study are available from the corresponding author upon reasonable request.
