# Supplementary material for: Balloon surface temperature–controlled ablation using a second-generation radiofrequency HotBalloon: an in vivo feasibility study
Source: Europace. 2023 Nov 9;25(12):euad340. doi: 10.1093/europace/euad340 (PMC10751809; doi:10.1093/europace/euad340)
Supplement: euad340_Supplementary_Data [file euad340_supplementary_data.zip › Supplmentary_table 2.pdf]

Supplementary Table 2: Electrophysiological and pathological outcomes in chronic evaluation

	57°C	60°C
Electrophysiological findings		
Acute electrical isolation		
Pulmonary vein isolation (%)	6/6 (100%)	6/6 (100%)
SVC isolation (%)	3/3 (100%)	3/3 (100%)
Total (%)	9/9 (100%)	9/9 (100%)
Chronic electrical isolation		
Pulmonary vein isolation (%)	3/6 (50%)	6/6 (100%)
SVC isolation (%)	2/3 (67%)	3/3 (100%)
Total (%)	5/9 (56%)	9/9 (100%)
PV/SVC stenosis		
>70% (severe)	1 (11%)	0 (0%)
50–70% (moderate)	1 (11%)	1 (11%)
Thrombus in balloon surface	0 (0%)	0 (0%)
Pathological findings		
Transmural ratio		
PV, n (%)	17/36 (47.2%)	31/39 (79.5%)
SVC, n (%)	19/20 (95%)	21/21 (100%)
All, n (%)	36/56 (64.2%)	52/60 (86.7%)
Lesion depth, µm		

PV, μm	1053 ± 1013	1208 ± 442
SVC, μm	952 ± 734	1076 ± 585
All, μm	1016 ± 906	1161 ± 494

Data are presented as mean ± standard deviation or as n (%).PV, pulmonary vein; SVC, superior vena cava.
