# Supplementary material for: Balloon surface temperature–controlled ablation using a second-generation radiofrequency HotBalloon: an in vivo feasibility study
Source: Europace. 2023 Nov 9;25(12):euad340. doi: 10.1093/europace/euad340 (PMC10751809; doi:10.1093/europace/euad340)
Supplement: euad340_Supplementary_Data [file euad340_supplementary_data.zip › Supplmentary table1.pdf]

Supplementary Table 1: Electrophysiological and pathological outcomes in acute evaluation

	51°C	54°C	57°C	60°C
Electrophysiological findings				
Injection volume				
PV, mL	9.6 ± 0.6	10.0 ± 0.7	9.6 ± 1.5	10.0 ± 1.2
SVC, mL	10.0	11.0 ± 1.4	11.0 ± 1.4	11.3 ± 2.5
Acute electrical isolation				
Pulmonary vein isolation (%)	0/2 (0)	3/6 (50)	5/5 (100)	6/6 (100)
SVC isolation (%)	0/2 (0)	2/2 (100)	3/3 (100)	3/3 (100)
Total (%)	0/4 (0)	5/8 (62.5)	8/8 (100)	9/9 (100)
Balloon surface thrombus (n)	0	0	0	0
Time to targeted BST, s	34.2 ± 13.6	70.4 ± 21.0	66.9 ± 25.2	69.2 ± 29.8
Mean BST, (°C)	50.7 ± 0.3	53.5 ± 0.5	57.0 ± 0.6	59.8 ± 0.8
Mean BCT, (°C)	58.4 ± 2.1	66.2 ± 4.8	70.8 ± 2.2	71.4 ±. 1.8
Mean RF power, (W)	53.8 ± 7.7	92.6 ± 11.6	105.3 ± 35.4	129.4 ± 33.4
Pathological findings				
Transmural ratio				
PV, n (%)	1/11 (9.9)	9/31 (29.0)	17/30 (56.7)	26/31 (83.8)
SVC, n (%)	5/18 (27.8)	8/12 (66.7)	9/12 (75)	12/12 (100)
All, n (%)	6/29 (20.6)	17/43 (39.6)	26/42 (61.9)	38/43 (88.4)
Lesion depth, µm				

PV, µm	948 ± 477	1009 ± 604	1255 ± 631	1562 ± 701
SVC, µm	639 ± 261	898 ± 323	1065 ± 219	1129 ± 480
All, µm	754 ± 379	975 ± 532	1201 ± 550	1418 ± 664

Data are presented as mean ± standard deviation or as n (%). BCT, balloon central temperature; BST, balloon surface monitoring; PV, pulmonary vein; RF, radiofrequency; SVC, superior vena cava.
