# Supplementary material for: Balloon surface temperature–controlled ablation using a second-generation radiofrequency HotBalloon: an in vivo feasibility study
Source: Europace. 2023 Nov 9;25(12):euad340. doi: 10.1093/europace/euad340 (PMC10751809; doi:10.1093/europace/euad340)

Supplementary Figure: Representative case of pulmonary vein stenosis. (**A**) Selective venography of the right superior pulmonary vein (RSPV) during energy application with a balloon surface temperature of 57°C. (**B**) Severe RSPV stenosis (yellow arrow) at 8 weeks after the ablation. RSPV, right superior pulmonary vein

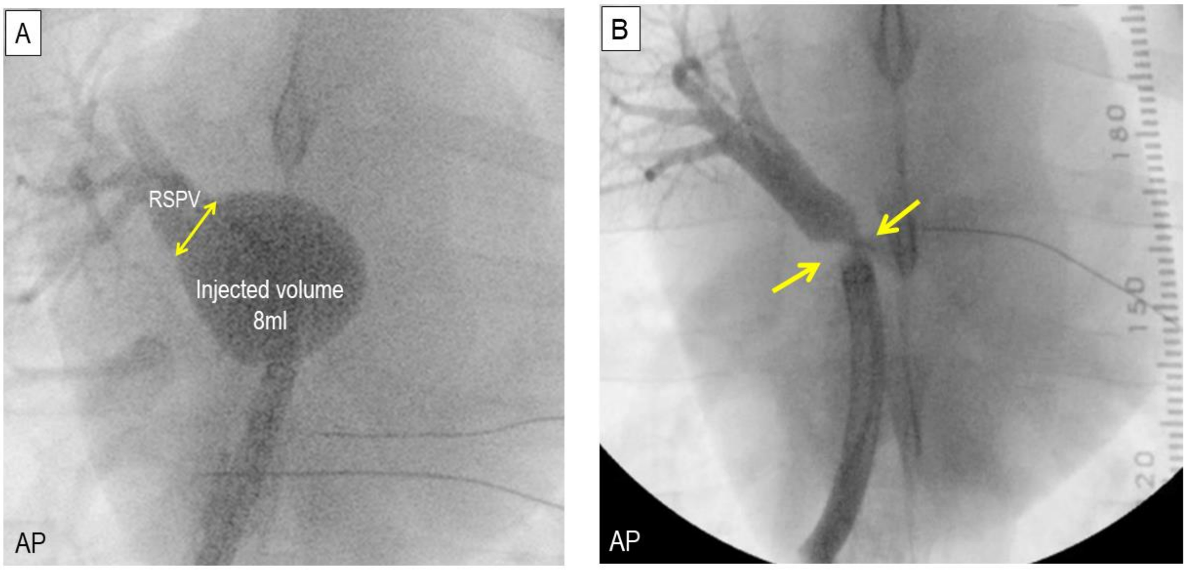

Supplement: euad340_Supplementary_Data [file euad340_supplementary_data.zip › Supplmentary_Figure.pdf]
